# Three-dimensional electron diffraction of nanocrystalline reticular materials: from structure to chemical insight

**DOI:** 10.1107/S2052252526011401

**Published:** 2026-11-09

**Authors:** Chiara Massera

**Affiliations:** a Department of Chemistry, Life Sciences and Environmental Sustainability University of Parma Parco Area delle Scienze 17/A Parma 43123 Italy

**Keywords:** three-dimensional electron diffraction (3D ED), metal–organic frameworks (MOFs), covalent organic frameworks (COFs), structural analysis

## Abstract

Three-dimensional electron diffraction is a powerful tool for studying nanocrystalline metal–organic and covalent organic frameworks, linking structure determination to chemical insight. In their topical review in this issue of *IUCrJ*, Wang & Huang [*IUCrJ* (2026), **13**, 675–688] highlight its role in discovering overlooked phases and revealing host–guest interactions, framework flexibility and structural dynamics.

A crystalline powder can conceal extraordinary architectures. Metal–organic frameworks (MOFs) and covalent organic frameworks (COFs) contain networks of building blocks surrounding pores that can capture gases, accommodate guests or support catalysis (Davis, 2002 ▸; Kitagawa, 2017 ▸; Côté *et al.*, 2005 ▸). MOFs consist of metal-containing nodes joined by organic linkers; COFs comprise organic building blocks connected by covalent bonds. For both, understanding their function requires more than knowing the chemical nature of their components; information about their three-dimensional arrangement and their response to incoming molecules and external stimuli is essential to unlock their full potential. However, many MOFs and COFs form crystals too small for conventional single-crystal X-ray analysis, and structure solution from powder X-ray diffraction can be extremely challenging. In their topical review in this issue of *IUCrJ*, Wang & Huang (2026 ▸) show how three-dimensional electron diffraction (3D ED), which enables the analysis of single nanocrystals, can overcome these drawbacks (Gruene *et al.*, 2018 ▸). In the past twenty years, 3D ED has experienced impressive developments in terms of hardware, software and methodology, being successful in the structure determination of a wide class of crystalline samples (Kolb *et al.*, 2007 ▸; Gemmi & Lanza, 2019 ▸; Gemmi *et al.*, 2019 ▸), including soft, beam-sensitive nanoporous materials (Huang *et al.*, 2021 ▸; Sun *et al.*, 2020 ▸). Wang & Huang highlight how this technique can not only determine the unknown structures of nanometre-sized crystals but also address broader chemical and structural questions, including the discovery of new materials and the investigation of host–guest interactions, framework flexibility and structural dynamics (Fig. 1 ▸).

A meaningful example discussed in the review is the discovery of the zeolitic imidazolate framework ZIF-EC1 (Ge *et al.*, 2021 ▸). This previously unknown MOF accounted for less than 5% of a multiphase powder. Analysis of more than 50 nanocrystals allowed its structure to be determined, and subsequent carbonization produced a material with better oxygen reduction activity than those derived from the other ZIFs tested. A phase that might otherwise have been overlooked proved to be a promising source of catalytic material.

Structural differences within an established framework family can be equally consequential, as in the case of COF-320 and COF-320-A, which show the same topology and degree of interpenetration but different imine-link orientations. The structures obtained by 3D ED helped explain why, under nitro­gen adsorption, COF-320 remained relatively rigid, whereas COF-320-A underwent several structural transformations (Kang *et al.*, 2023 ▸).

Other studies in the review show the effectiveness of 3D ED in determining layer stacking, locating molecules within pores and following solvent removal. These measurements help to answer questions central to porous-material chemistry: which phases have formed, where molecules bind and how the framework accommodates them.

There are boundaries to this progress. Beam damage, limited crystallinity and incomplete diffraction data remain obstacles. Multiple scattering can cause measured intensities to depart from those predicted by the kinematical approximation, requiring a dynamical treatment to improve the accuracy of the refined structural model. Microscope vacuum, temperature and sample preparation may alter a guest-containing framework; structures measured under these conditions must be related carefully to adsorption, spectroscopy and performance measurements for reliable chemical interpretation. On the other hand, further automation and improved data analysis should make 3D ED more widely usable, while measurements under controlled gas or liquid environments could help to establish how framework structures change during use, helping to connect synthesis and structure with material properties.

The present review offers an opportunity to assess both progress and the work ahead. Ultimately, 3D ED enables crystalline powders of MOFs and COFs to yield detailed structural information, connecting phase identification with an understanding of framework properties and behaviour.

## Figures and Tables

**Figure 1:**
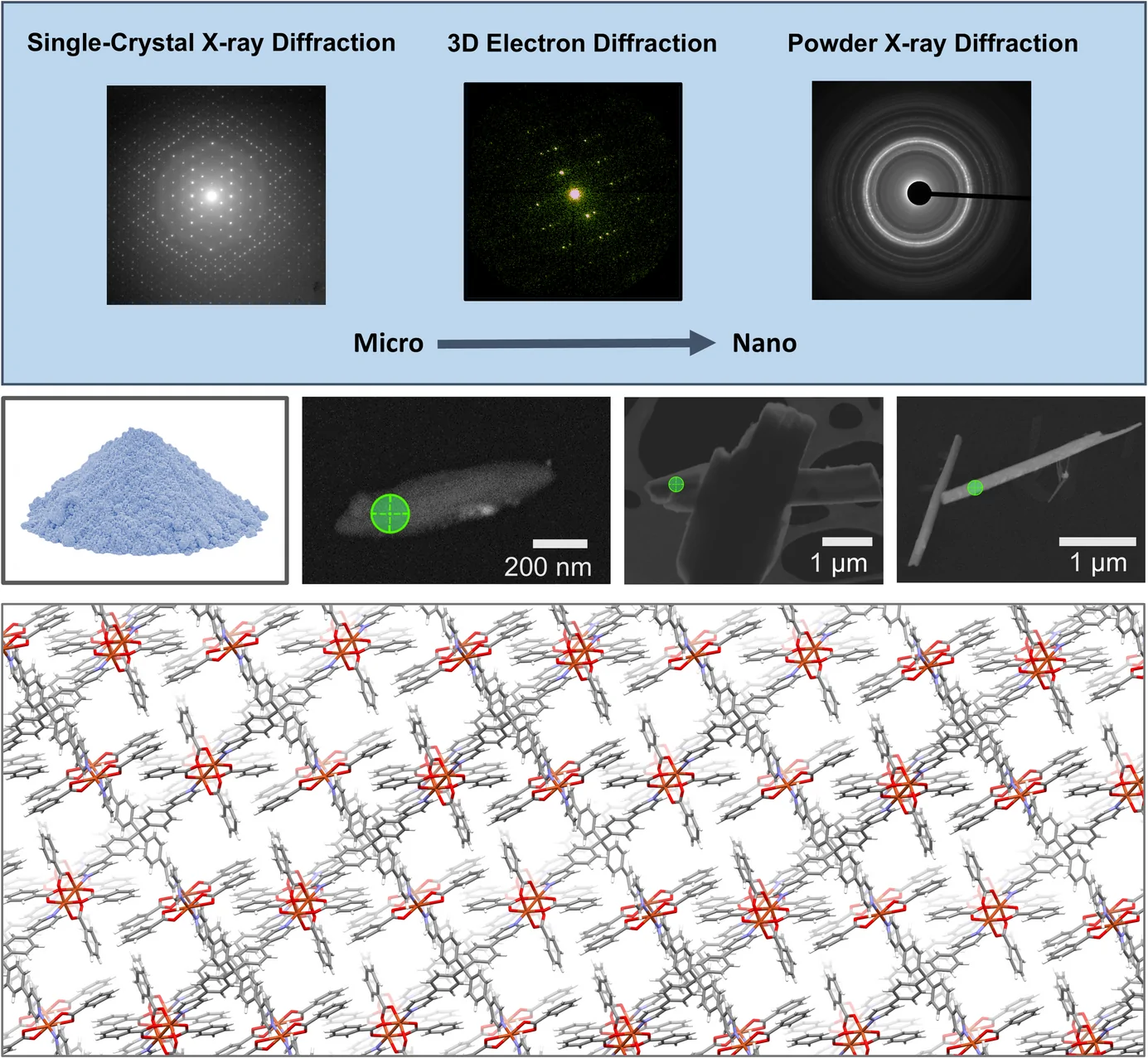
3D ED is a powerful tool complementing single-crystal and powder X-ray diffraction, allowing one to perform ‘local’ or ‘nano’ diffraction experiments. A nanometre-sized parallel electron beam is employed to collect data on specific regions of different nanocrystals. Structure solution and analysis can then reveal framework connectivity, guest binding, changes in pore geometry and responsive behaviour.
